# Experiences of children who have been separated from a parent due to military deployment:a systematic review of reviews

**DOI:** 10.1192/bjo.2021.702

**Published:** 2021-06-18

**Authors:** Jennifer Kent, Pamela Taylor, Sarah Argent, Narasha Kalebic

**Affiliations:** Cardiff University

## Abstract

**Aims:**

To conduct a systematic review of reviews to investigate how military deployment of a parent affects his/her child, and the extent to which the child's own perspectives have been documented.

**Background:**

Lengthy but finite disruptions to parenting in any form may affect child development and mental and physical health.

Military deployment means weeks or months of separation from one parent.

2016 figures for the U.S. military showed that 40.5% of military personnel have children, and of these 1.7 million children the largest percentage are aged between 0–5 years (37.8%).

**Method:**

Seven databases were searched: AMED, Web of Science, Scopus, EMBASE 1947, Joanna Briggs Institute EMP database, Ovid MEDLINE 1946 and PsycINFO 1806 from the inception of each electronic database until 31st March 2018.

Inclusion criteria:

Child and young adults aged 0–24 years

English language papers only

All papers being systematic reviews or meta-analyses

A focus on documenting the effects on child outcomes

Data extracted included the review methods and child outcomes reported, including educational attainment; physical symptoms; mental illnesses or disorders; changes to behaviours, and effects on peer and parental relationships.

**Result:**

The eight reviews identified included 32 common and relevant studies.

Across the various studies, only about 20% of data came directly from children.

Five papers extracted from the reviews identified parental deployment as having a negative effect on school attainment.

Nine studies extracted from the review papers found a positive correlation between having a deployed parent and a greater chance of experiencing depressive symptoms and feelings of anxiety.

Strong correlations of increased prevalence of both externalising & internalising behaviours were conclusively found in 7 of the reviews.

Increased resilience was detailed in only one study featured in multiple reviews.

Just one study featured across the reviews reported on physiological measures - adolescents with deployed parents had higher blood pressures and significantly higher heart rates and stress scores than civilian children.

**Conclusion:**

More research obtaining the viewpoint of the child directly and observation of such children is required to properly understand the effects on children with a deployed parent, without the interference of parent or teacher reporting bias. Additionally, with only one study reporting on increased offspring resilience there has been limited exploration of potential positive correlates, so further research regarding these is important.

